# Unusual anatomical variations of cervical and cranial arteries with surgical interest: case report

**DOI:** 10.1590/1677-5449.202400482

**Published:** 2025-05-30

**Authors:** Monique Boukobza, Jean-Pierre Laissy

**Affiliations:** 1 Paris University, Paris, France.; 2 Bichat-Claude Bernard Hospital, Assistance Publique Hôpitaux de Paris, Department of Radiology, Paris, France.

**Keywords:** type 2 persistent primitive proatlantal artery, left common carotid artery variation, extracranial internal carotid artery aneurysm, vertebral artery hypoplasia, kinking, azygos anterior cerebral artery, artéria proatlantal primitiva persistente tipo 2, variação da artéria carótida comum esquerda, aneurisma extracraniano da artéria carótida interna, hipoplasia da artéria vertebral, torção, artéria cerebral ázigos anterior

## Abstract

We report an exceptional case of a large right persistent proatlantal artery (PPPA), defined as a “mixed” type, because it originated as in Type 2 from the external carotid artery and courses as in Type1, i.e. over the vertebral artery groove of the posterior arch of C1, entering the skull via the foramen magnum, without passing through the transverse foramen of any of the cervical vertebra. This “mixed” PPPA was associated first with an azygos anterior cerebral artery and second with a left common carotid artery arising from the brachiocephalic trunk. The right occipital artery arose from the PPPA. In addition, an aneurysm arose from the kinked right cervical internal carotid artery. This case illustrated an association not reported previously, making treatment of the concomitant aneurysm challenging. Moreover, understanding of persistent carotid-vertebral anastomosis is essential to enable evaluation and management before performing endovascular treatment, especially in cases of basilar thrombectomy and for posterior circulation strokes.

## Resumo

Relatamos um caso excepcional de uma grande artéria proatlantal persistente (APP) direita de tipo “misto”, pois origina-se como tipo 2 da artéria carótida externa e segue como tipo 1, ou seja, passa sobre o sulco da artéria vertebral no arco posterior de C1, entrando no crânio através do forame magno, sem atravessar o forame transverso de qualquer vértebra cervical. A APP “mista” foi associada, em primeiro lugar, a uma artéria cerebral anterior ázigo e, em segundo, a uma artéria carótida comum esquerda proveniente do tronco braquiocefálico. A artéria occipital direita originou-se da APP2. Além disso, um aneurisma surgiu da artéria carótida interna cervical direita dolicho. Este caso revela uma associação inédita, o que representa um desafio no tratamento do aneurisma concomitante. A compreensão da anastomose carótida-vertebral persistente é essencial para a avaliação e manejo antes de um tratamento endovascular, especialmente em casos de trombectomia basilar e de acidentes vasculares cerebrais na circulação posterior.

## INTRODUCTION

The type 2 persistent primitive proatlantal artery (PPPA2) is a rare variant of the persistent carotid-vertebrobasilar anastomoses. Because the initial segment of the ipsilateral VA is in most cases aplastic or hypoplastic, the PPPA2 is large. We report a case in which a very large PPPA2 with an absent ipsilateral vertebral artery (VA) was found incidentally on imaging.

The patient also presented an aneurysm of the external segment of the ipsilateral internal carotid artery (ICA), dolichoectasia, an azygos anterior cerebral artery (ACA), and a left common carotid artery (CCA) arising from the brachiocephalic trunk (BCT).

## CASE REPORT

A 63-year-old man presented acute onset of motor aphasia related to a middle cerebral artery territory (MCA) infarct. An occlusion of the MCA M2 segment was diagnosed on MR angiography.

Computed tomography angiography (CTA) revealed a right PPPA2 branching from the dorsal part of the external carotid artery (ECA) at the level of C1-C2. This right ECA presented a marked enlargement compared to the left one: 18mm vs. 3.3 mm at the C3-C4 level and 9 mm vs 3.6 mm at the C2 level. The right common carotid artery (CCA) was also enlarged: maximum diameter of 19.5mm vs. 10.2 for the left one.

**Figure 1:**
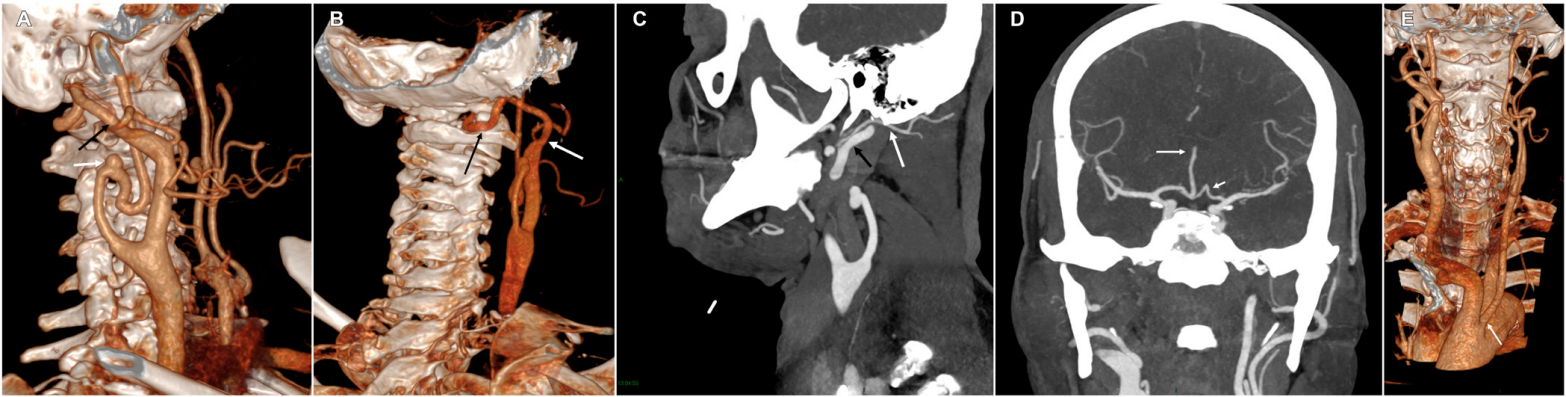
**A)** Volume-rendering CT angiography shows the type 2 persistent primitive proatlantal artery (PPPA2) arising from a large right external carotid artery (ECA) (black arrow) and a wide-necked saccular aneurysm (5.4 x 6.4 mm in the axial plane) arising from the kinked right internal carotid artery (ICA) at the C3 level (white arrow). **B)** Volume-rendering CT angiography shows the type 2 persistent primitive proatlantal artery (PPPA2) coursing over the right vertebral artery groove of the posterior arch of C1 (black arrow). Note the origin of the PPPA2 from the right external carotid artery (white arrow). **C)** Multiplanar reformation (MPR) from the CT angiography shows the right occipital artery (white arrow) arising from the right type 2 persistent primitive proatlantal artery (PPPA2) (black arrow). **D)** Multiplanar reformation (MPR) from the CT angiography shows an azygos anterior cerebral artery (long arrow) and the kinked A1 segment of the left anterior cerebral artery (short arrow). **E)** The left anterior oblique projection of volume-rendering CT angiography shows the origin of the left common carotid artery (CCA) that arises from the brachiocephalic trunk (BCT) (arrow).

This right PPPA2 coursed upward and very externally. It then took a dorsal course, described two loops, passed over the posterior arch of the atlas, and entered the skull via the foramen magnum (Figures 1A and 1B). The PPPA2 remained as a basilar artery, which ended in posterior cerebral arteries. It did not course through the transverse foramen of any cervical vertebra. This PPPA gave rise to the right occipital artery (Figure 1C).

CTA also showed a wide-necked saccular aneurysm (5.4 x 6.4 mm, maximal diameter in the axial plane) arising from the kinked right ICA at the C3 level (Figures 1A and 1B). The right CCA was also very large.

The right VA was absent and the left one was very hypoplastic and terminated its course at theC3 level. The right PPPA was the exclusive blood supply of the posterior circulation as neither of the posterior communicating arteries were visualized.

In addition, an azygos anterior cerebral artery, kinking (level 3) of the left anterior cerebral artery (ACA) A1 segment (Figure 1D), and the left CCA arising from the BCT were also observed (Figure 1E). There was bilateral kinking (grade 1) of both ICAs, without stenosis, at the C2-C3 level (Figure 1E). No intracranial aneurysm and no atheroma were depicted. Conservative management with follow-up imaging were chosen.

## DISCUSSION

The proatlantal intersegmental artery is one of the carotid-basilar anastomoses of the embryonic period, which persist until VAs develop (sixth gestational week). When the PPPA fails to degenerate normally, this vessel forms an anastomotic branch between the ICA and the vertebrobasilar system.

The PPPA2 is a rare cerebral artery variant, with an incidence of approximately 0.02%,^1^ which arises from the ECA at the level of the C2-C3 vertebra, usually joins the VA between the atlas and axis, passes through the transverse foramen of the atlas, and enters the skull via the foramen magnum. Bilateral PPPA2 is an exceptional occurrence.

When the PPPA2 is large, the proximal VAs are usually hypoplastic, and the ipsilateral VA may be absent. In addition, the PPPA is often accompanied by posterior communicating artery hypoplasia. In these conditions, posterior circulation depends on the PPPA, and the PPPA2 may be associated with a higher incidence of posterior ischemic events. In the present case there was no ipsilateral VA and the contralateral VA was highly hypoplastic. Thus, the PPPA2 supplied the majority of the blood to the posterior circulation. The PPPA2 arose from an enlarged ECA at the level of the C1-C2 intervertebral disk, in a patient presenting a greatly enlarged CCA. The course of this PPPA2 is similar to that described in the Ma et al.^2^ case, i.e., a PPPA originating as a Type 2 from the ECA and coursing as a Type 1 over the VA groove of the posterior arch of C1, which they called a “mixed PPPA”.

A few cases of PPPA (type 1, 2, or mixed) have been reported with concomitant ICA/VA stenosis, basilar occlusion, or vertebra-basilar aneurysm, which were treated by an endovascular approach. Recently, Lin et al.^3^ reported angioplasty and intracranial stenting of a V4 segment (intracranial segment of the VA) stenosis via a contralateral PPPA2, and Ito et al.^4^ reported a mechanical thrombectomy for basilar occlusion via a left PPPA1, the right VA being hypoplastic.

Understanding of persistent carotid-vertebral anastomosis is essential to enable evaluation and management before performing an endovascular treatment for posterior circulation atherosclerotic stenosis and basilar thrombectomy. Thus, preoperative CT angiography (CTA) examination is particularly important to assess persistent carotid-vertebral anastomosis, in order to prevent or avoid difficulties in identifying effective catheter access during emergency thrombectomy in case of acute occlusion of the basilar trunk.

The azygos anterior cerebral artery (azygos ACA) is a rare arterial variant characterized by an absent anterior communicating artery with the two ACAs joined in their proximal segments (horizontal segment, A1), forming a single-trunk vertical A2 segment - the unpaired ACA - arising through the interhemispheric fissure and supplying the medial surface of both hemispheres. Baptista^5^ proposed a 3-type classification of unpaired ACAs. Type I, “the true azygos ACA”, has a single unpaired ACA supplying the medial surface of both cerebral hemispheres. Type II, the “bi-hemispheric ACA”, has both right and left ACAs. Type III, “the ACA trifurcation”, has a third artery, i.e. the accessory ACA arising from the anterior communicating artery. The patient reported here has a Baptista Type 1 azygos ACA, which is explained from an embryological point of view by the non-differentiation of two A2 segments during Padget’s Stage V (16-18mm).^6^

Persistent primitive arteries, azygos ACA, and fenestration of the intracranial arteries are more often associated with intracranial aneurysms than other vessels. To date, intracranial aneurysms have been reported associated in 6 cases of PPPA1,^7^ and in 2 cases of PPPA2.^7,8^

Extracranial ICA (EICA) aneurysms are very uncommon and account for less than 1% of all arterial aneurysms and for less than 1% of all carotid pathologies.^9^ Any of the segments of the ICA can be involved, although the mid to distal EICA is the most commonly affected. Carotid tortuosity is associated with EICA aneurysms and seems to be a risk factor for the development of such aneurysms.^9^ EICA aneurysms may lead to neurologic symptoms including transient ischemic attacks (TIAs) or ischemic stroke. A recent study showed that in patients with asymptomatic EICA aneurysms, the risk of cerebral infarction is small but not minor and that conservative management seems justified, in particular in patients without growth.^10^ There is no clear treatment algorithm for these aneurysms.^11^ In the present patient, conservative treatment was chosen with follow-up imaging. Neither the EICA or the PPPA were responsible for the stroke.

Among patients with multiple cerebral arterial variations occasionally reported, this is the first report of concomitant PPPA and EICA aneurysm. This combination makes treatment of the concomitant aneurysm challenging from both an endovascular and an open surgery perspective. Understanding of persistent carotid-vertebral anastomosis is essential to enable evaluation and management before performing endovascular treatment, especially in cases of basilar thrombectomy and for posterior circulation strokes.

This manuscript is in accordance with the Helsinki Declaration and with the institutional (Bichat-Claude Bernard Hospital, Paris) ethical guidelines.

Informed consent was obtained
